# Challenges in recruiting and retaining adolescents with abuse-related posttraumatic stress disorder: lessons learned from a randomized controlled trial

**DOI:** 10.1186/s13034-020-00320-y

**Published:** 2020-04-16

**Authors:** Anna Vogel, Hannah Comtesse, Rita Rosner

**Affiliations:** Department of Psychology, Catholic University Eichstaett-Ingolstadt, Ostenstrasse 25, 85071 Eichstaett, Germany

**Keywords:** Posttraumatic stress disorder, Adolescents, Young adults, Recruitment, Barriers, Facilitators, Study participation

## Abstract

**Background:**

Research on effective recruitment and retention strategies for adolescents and young adults suffering from posttraumatic stress disorder is scarce. The aim of the current study was to provide data on recruitment sources, barriers, and facilitators for participation in a randomized controlled trial for young individuals with histories of sexual and/or physical abuse.

**Methods:**

Study participants aged 14 to 21 were asked to complete a checklist on individual sources of recruitment, barriers, and facilitators for participation in the trial. Fifty-three out of the 80 study participants who were contacted completed the checklist (66.3%).

**Results:**

Most respondents reported multiple recruitment sources, with online and media advertising search strategies indicated most frequently (45.4% of all mentions), followed by practitioner-referred sources (38.7%). Respondents’ reported barriers included additional demands of the trial (60.3%), followed by distress caused by having to talk about painful topics (15.5%). The most frequently indicated facilitators were the organizational setting (55.1%) and monetary incentives (22.2%), followed by social support (12.0%) and non-monetary incentives (10.2%). No significant differences were observed between adolescent and young adult respondents with the exception that adolescents reported significantly more frequently that they had learned about the trial from their caregiver.

**Conclusions:**

Our findings permit the formulation of recommendations for planning and conducting trials with this clientele. Future research is needed on how specific barriers can be effectively overcome.

## Background

### Strategies for improving recruitment to randomized controlled trials

Implementing randomized controlled trials (RCTs) is associated with numerous challenges. Poor recruitment in particular is a widespread problem, with many trials failing to reach their planned sample size within the planned timescale [1–4]. Failure to meet the recruitment goals increases the risk of trials being underpowered [1], closed prematurely, or needing extended recruitment periods, including increased costs and workload [2, 5]. Poor recruitment, therefore, increases the risk of delaying the implementation and dissemination of potentially effective interventions [see 5]. To tackle these problems, several studies sought to identify the methods used to improve recruitment of participants [e.g., 5, 6] and clinicians [e.g., 1, 6, 7] as well as successful trial design and procedure features [e.g., 2, 6, 8, 9]. Overall, the results were inconclusive and no single study was included that specifically examined interventions for posttraumatic stress disorder (PTSD).

### Barriers and facilitators for recruitment to mental health trials

A systematic review [10] identified several barriers to adult patient participation in RCTs, such as additional demands on the patient (e.g., additional appointments, travel problems and costs), patient preferences for a specific treatment, worries about the uncertainty of treatment efficacy, and concerns about information and consent. In the context of research on the use of mental health services, several barriers specifically relevant to ethnic minority participants have been added, such as individual explanatory models of illness, help-seeking behavior, stigma (for self and family), medical insurance, lack of childcare, and language difficulties [11, 12]. However, most of them are not unique to participants from ethnic minorities, although the way in which they manifest themselves might often be distinct in different minority groups.

### Challenges for clinical research with participants suffering from PTSD

Despite the severe consequences of untreated PTSD for both individual mental health and society [13, 14], a significant proportion of treatment-seeking individuals suffering from PTSD do not initiate trauma-focused treatments [15] despite their efficacy in reducing PTSD symptoms [16–18]. Individuals with PTSD have been shown to endorse more barriers to mental health treatment than individuals with anxiety disorders [19]. Furthermore, individuals with PTSD have an increased risk of dropping out of treatment [20], particularly in the case of elevated symptoms of PTSD-typical avoidance [21]. Thus, reaching and retaining individuals suffering from PTSD constitutes a specific challenge for mental health research.

A recent meta-analysis of adult trauma survivors [22] revealed that the most prominent barriers to mental health service utilization include concerns related to stigma, shame, and rejection; low mental health literacy (i.e., not believing that symptoms are part of mental illness); lack of knowledge and doubts about services; fear of negative social consequences (e.g., on career); limited resources with respect to access, language, time, and financial barriers; and trauma-specific barriers (i.e., not wanting to talk about the trauma). According to the authors, facilitators were only investigated in a small number of studies with social support, a desire for change, and prior positive experiences with professional help being reported by several primary studies. Other facilitators in adults suffering from PTSD might be associated with logistic issues and the organizational setting [15]. Accordingly, patients might be enabled to initiate trauma-focused treatments not only because of their own ability to time treatment according to already existing priorities and responsibilities, but also because of the specific type of referral source (with mental health care referral sources being more enabling than primary care referral source), and the availability and consistency of trained providers of trauma-focused treatments. These PTSD-specific factors must be taken into account when planning therapy research with this clientele.

### Challenges for clinical research with abused adolescents and young adults

With regard to trauma-exposed adolescents, the number of individuals who refrain from seeking professional help is particularly high [23]. Given the influence that striving for autonomy may have on engagement in the therapeutic process and on the therapeutic alliance, the developmental transitions in adolescence might impact the willingness and the ability to engage in and to complete treatment (for abuse-related PTSD, [24], for anxiety disorders in general, [25]). A recent analysis of 3081 trauma-exposed adolescents aged 12 to 16 [23] identified a percentage of 43.5 who had very low probabilities of service usage across the mental health, juvenile justice, social services, school and healthcare systems. These data indicate that recruitment for mental health research in this clientele might be particularly difficult.

Although there is a helpful compilation of advisable recruitment and retention strategies for young study participants in a broader age range (3–18 years, [26]), data on effective recruitment sources that specifically focus on adolescents and/or young adults are scarce. With regard to participation in mental health trials, eligible adolescents suffering from depression or anorexia who were referred by a professional (e.g., health care provider), were more likely to give their assent than those recruited by advertisement, regardless of other factors [27, 28]. Other studies reported on the potential of the social media [29] and various electronic sources [30] for recruiting adolescents. To our knowledge, no data on successful recruiting strategies for adolescents and young adults suffering from PTSD have been published so far.

One particular challenge in the recruitment of underage study participants is obtaining parental permission in addition to the adolescents’ assent. Even though in most countries adolescents are allowed by law to independently obtain medical treatments without the consent of a parent or legal guardian [31], participation in a psychotherapy study requires the informed consent of one, often all, parents or legal guardians for ethical reasons. This can be an additional barrier—for example if (a) the research focuses on sensitive issues that the adolescent cannot or does not want to reveal to the legal guardian, such as abuse or substance use [32, 33]; if (b) there are age-related conflicts of autonomy between the adolescent and the legal guardians and this has an effect on the refusal of a study consent by one of the parties; or if (c) parents or legal guardians are critical of psychotherapy in general or of participation in scientific trials in particular due to their own problems or previous experiences [34, 35]. Furthermore, it has been shown that more abstract issues relevant to participation in clinical trials (e.g., understanding the primary purpose of the trial or the probability of receiving a control treatment) are not well understood by children, adolescents, and many parents [36]. Moreover, especially if there are only limited services available for particularly vulnerable populations, researchers and Institutional Review Boards (IRBs) are faced with the question of how to recruit for a clinical trial while complying with all ethical principles. These issues confront researchers with ethical challenges.

Retention poses a significant challenge for psychotherapy research with abused adolescents and emerging adults. Generally speaking, a large proportion of treatments in youth mental health care results in premature termination (28–50%, [37]). Furthermore, there is a considerable rate of attrition from clinical research trials (e.g., 35% among children aged 7–12 years enrolled in a study on group psychotherapy for youth with prominent psychosocial deficits, [38]). Specifically, there is some evidence that older children and adolescents are more likely to terminate trauma-focused treatment prematurely [39–41] and tend to be less likely early-responders [42] than younger individuals. However, the role of parents and legal guardians is not limited to giving consent to study. Up into young adulthood, they are considered as important partners for communication from the first recruitment to the regular participation in study appointments [43, 44]. Thus, it does not seem surprising that a high parental psychopathological burden is associated with their children’s premature termination of treatment [45]. Despite these alarming results, no evaluation of effective retention strategies for adolescents and young adults suffering from PTSD or for their parents or legal guardians exist to date.

### The need for data on recruitment and retention of adolescents and young adults suffering from abuse-related PTSD

While there are numerous studies that examine treatments for adults with PTSD (for an overview, see [46, 47]) and children and adolescents in a broader age range suffering from PTSD (for an overview, see [48–50]), there are very few controlled trials focusing exclusively on adolescents and/or young adults (for an overview, see [51]). This is especially the case when focusing on child sexual and/or physical abuse–related PTSD in adolescents and/or young adults despite a growing body of evidence that PTSD, after histories of multiple traumas in childhood, tends to be associated with higher levels of symptom complexity [52, 53] or severity [54]. To our knowledge, only two RCTs have been conducted that were tailored specifically to adolescents suffering from abuse-related PTSD [55, 56]. Furthermore, sample sizes were small given the respective timescales for screening in both RCTs (*N* = 61 in 6 years, [55], *N* = 88 in 2 years, [56]). These numbers also indicate that research with adolescents suffering from abuse-related PTSD might be challenging. To facilitate the conduct of future trials, it is important to examine recruitment and retention strategies that work in adolescents and young adults with PTSD after abuse. However, no study has reported strategies for this specific clientele up to now. In the present study, we were explicitly interested in data concerning young adults as well. Even if there are numerous adult studies including young adults, the generalizability of results from PTSD treatment research to young adults remains unclear because of the lack of information on refined age reporting in clinical trials (see [51]). Furthermore, it might be helpful to consider developmental aspects in treatment and clinical research with regard to young adults as well. This is because they are still in a dynamic, sensitive period of ongoing biopsychosocial development within an extended transition period to adulthood in today’s society, and psychopathologies, such as PTSD, may lead to delays in psychosocial development [57, 58]. Therefore, the recruitment and retention of young adults might be different than that of older adults.

The aim of this study was to provide insight into promising recruitment methods, barriers, and facilitators to a RCT for young study participants with PTSD after histories of sexual and/or physical abuse. We aimed to examine recruitment methods, barriers and facilitators for study participation reported by study participants aged 14 to 21 and whether there were differences between adolescent and young adult participants. Given the exploratory design of this study, there were no a priori hypotheses.

## Methods

### Participants and procedure

Participants aged 14 to 21 years suffering from PTSD after sexual and/or physical abuse were recruited for a multicenter RCT to evaluate developmentally adapted Cognitive Processing Therapy [D-CPT, 24] in comparison to a wait-list condition with treatment advice (WL/TA) at three university outpatient mental health clinics in Germany from July 2013 to June 2015 [56]. Health insurance covered the psychotherapy costs. A range of clinical interviews and questionnaires were assessed at baseline, posttreatment and 3-month follow-up. The study protocol was approved by the IRBs of the Catholic University Eichstätt-Ingolstadt, Eichstätt, Germany; the Freie Universitaet of Berlin, Berlin, Germany; and the Goethe University Frankfurt, Frankfurt am Main, Germany (for the study protocol, see [59]; German Clinical Trials Register identifier: DRKS00004787). Written informed consent was obtained from all adult participants and from parents or guardians of minors. In addition, written assent was obtained from all minor participants using the same study information as for adult participants. During the recruitment phase of the trial a variety of strategies had been implemented to inform possible study participants about the trial and to enhance participants’ retention. Information on all strategies applied can be obtained in Additional file 1.

In this study, we re-contacted study participants for a follow-up survey between May 2016 and July 2016, i.e., after already having completed the 3-month follow-up. Participants were asked to complete a checklist on individual sources of recruitment, barriers and facilitators for participation in the trial. All participants were first contacted via letter, including a cover letter, the checklist and a prepaid return envelope. The cover letter included a link and QR code to an online version of the checklist, if participants preferred this to the paper–pencil checklist. If participants did not reply within 3 weeks, they were sent a reminder via email or phone. There was no financial compensation for returning the checklist. In this study, we contacted all participants included in the per protocol analysis (*N* = 80), as there were 8 participants who had been excluded due to violation of eligibility criteria disclosed during treatment. We were unable to locate nine participants, and a further 18 did not respond to our survey, resulting in a response rate of 66.3% (*n *= 53).

### Measures

Sociodemographic and trauma-related information was obtained during the baseline assessment of the RCT, including age, gender, immigration background, current living circumstances, employment, family status, and trauma type (physical and/or sexual abuse).

A checklist was developed to assess participants’ individual recruitment sources, barriers, and facilitators for study participation. Each of the three parts of the checklist (1: recruitment sources, 2: barriers, 3: facilitators) began with a question and a short introduction. The items in the three parts of the checklist were generated on the basis of applied recruitment and retention efforts and literature regarding relevant frequent recruitment sources [26], facilitators, and barriers [12]. We also added some barriers we had come across in the reports of our study participants: video recordings, volume of questionnaires, and alternating interviewers in diagnostic assessments. The respective parts of the checklist included 27 different recruitment sources, nine different barriers, and 10 different facilitators. Participants were asked to indicate all applicable answers. There was also space to provide answers other than those listed. Consequently, there was no maximum number of possible chosen items. See Additional file 2 for the checklist.

The severity of PTSD symptoms was measured with the Clinician-Administered PTSD Scale for Children and Adolescents (CAPS-CA, [60], German version [61]. This structured clinical interview rates the frequency and intensity of PTSD symptoms on a scale ranging from 0 (*never/no problem*) to 4 (*most of the time/extreme*). The severity score was calculated by summing all the frequency and intensity scores of the 17 symptom questions according to the Diagnostic and Statistical Manual of Mental Disorders, Fourth Edition (DSM-IV, [62]). Possible scores ranged from 0–136. For this study, we calculated changes in CAPS-CA scores from baseline to the 3-month follow-up.

Comorbid mental disorders according to DSM-IV criteria were assessed using the Structured Clinical Interview for DSM-IV Axis I (SCID-I, [63], German version [64]), together with the borderline section of the Structured Clinical Interview for DSM-IV Axis II ([65], German version [66]). The presence of typical childhood mental disorders which are not covered by the SCID-I, was determined using specific modules of the Diagnostic Interview for Mental Disorders in Children and Adolescents [67]. The current or lifetime comorbid diagnosis of nicotine abuse was assessed using the corresponding module of the Expert System for Diagnosing Mental Disorders [68]. In this study, we report on the number of comorbid disorders fulfilled at baseline and at the 3-month follow-up.

### Statistical analysis

#### Quantitative data analysis

Differences between respondents and non-respondents in terms of sociodemographic, trauma and outcome variables were computed using χ^2^ tests, Fisher’s exact tests (in the case of small cell sizes) and *t*-tests. To investigate the frequency of reported recruitment sources, barriers, and facilitators, we counted the number of chosen items in each part of the checklist. Differences between adolescents (aged 14 to 17) and young adults (aged 18 to 21) in terms of the reported frequencies of chosen items in the checklist were calculated using χ^2^ tests and, in the case of small cell sizes, Fisher’s exact tests. The significance level for all analyses was set to 0.05 (2-tailed).

#### Qualitative data analysis

The answers given in the checklist in an open format on participants’ individual recruitment sources, barriers, and facilitators were grouped by content and assigned to existing categories covered by recruitment sources, barriers, and facilitators already included in the checklist, or new categories, using content analysis method [69]. Two researchers (including the first author) undertook the assignment to the categories independently of each other. In individual cases of divergence, consensus had to be achieved. Of the 10 answers given in an open format with regard to recruitment sources, four were assigned to existing categories (internet search: *n* = 3; parent/caregiver: *n* = 1) and six were grouped in two new categories (close friends: *n* = 2; other: *n* = 4). Of the nine answers given in an open format with regard to barriers to study participation, one was subsumed under an existing category (alternating interviewers: *n* = 1) and seven were grouped in three new categories (difficulties in scheduling appointments: *n* = 3; distress caused by using public transportation to the study site: *n* = 2; other: *n* = 3). Of the seven answers given in an open format regarding facilitators for study participation, one was assigned to an existing category (flexible time scheduling: *n* = 1) and six were grouped in two new categories (empathy of study staff: *n* = 4; other: *n* = 2).

## Results

### Sample characteristics

The respondents’ ages ranged from 14 to 21, with *n* = 25 aged under 18; 90.2% were female. Please refer to Table 1 for information on sociodemographic, trauma, and outcome characteristics of all respondents and non-respondents. Two questionnaires could not be matched to their respective identification number, so we were not able to report any information on the respective characteristics of these respondents and excluded these questionnaires from all analyses. The respondents had been randomized to D-CPT (47.1%) and the WL/TA condition (52.9%) in nearly equal proportions. The response rates at the respective study sites were very similar (*n*_Frankfurt_ = 28.3%; *n*_Berlin_ = 32.1%; *n*_Ingolstadt_ = 35.8%). Forty-four respondents completed the paper–pencil version, while nine used the online version of the checklist.

**Table 1:** Sociodemographic, trauma, and outcome characteristics

Characteristic	Study population			*p* value
	All (*N* = 88)	Respondents (*n* = 51^a^)	Non-Respondents (*n* = 37)	
Female, No. (%)	75 (85.2)	46 (90.2)	29 (78.4)	0.12^b^
Age in years, *M* (*SD*)	18.12 (2.24)	17.96 (2.21)	18.34 (2.29)	0.44^c^
Immigration background, No. (%)	23 (26.1)	13 (25.5)	10 (27.0)	0.87^b^
Out-of-home placement or institutional care, No. (%)	25 (28.4)	14 (27.5)	11 (29.7)	0.82^b^
Support by youth welfare services, No. (%)	21 (23.9)	7 (13.7)	14 (37.8)	0.01^b*^
Occupation status, No. (%)				
Employed	3 (3.4)	2 (3.9)	1 (2.7)	1.00^d^
Unemployed	8 (9.1)	4 (7.8)	4 (10.8)	0.63^b^
In training/education	72 (81.8)	44 (86.3)	28 (75.7)	0.20^b^
Other	5 (5.7)	1 (2.0)	4 (10.8)	0.16^d^
History of sexual abuse, No. (%)	70 (77.8)	39 (73.6)	31 (83.8)	0.40^b^
History of physical abuse, No. (%)	72 (80.0)	43 (81.1)	29 (78.4)	0.48^b^
Randomized into D-CPT, No. (%)	44 (50.0)	24 (47.1)	20 (54.1)	0.52^b^
Study site Berlin	13 (29.5)	8 (33.3)	5 (25.0)	0.55^b^
Study site Frankfurt	17 (38.6)	8 (33.3)	9 (45.0)	0.43^b^
Study site Ingolstadt	14 (31.8)	8 (33.3)	6 (30.0)	0.81^b^
Interviewer-rated PTSD at baseline, CAPS-CA^e^, *M* (*SD*)	65.57 (22.13)	63.37 (20.27)	68.59 (24.42)	0.27^c^
Change of interviewer-rated PTSD from baseline to 3-month follow-up, CAPS-CA^fg^, *M* (*SD*)	28.55 (25.22)	28.73 (25.13)	28.15 (26.08)	0.93^c^
Comorbid DSM-IV disorders at baseline^h^, No (%)				
0	18 (20.5)	13 (25.5)	5 (13.5)	0.17^b^
1 or 2	41 (46.6)	21 (41.2)	20 (54.1)	0.23^b^
≥ 3	29 (33.0)	17 (33.3)	12 (32.4)	0.93^b^
Comorbid DSM-IV disorders at 3-month follow-up^hi^, no (%)				
0	21 (23.9)	17 (33.3)	4 (10.8)	0.17^b^
1 or 2	27 (30.7)	18 (35.3)	9 (24.3)	0.66^b^
≥ 3	18 (20.5)	11 (21.6)	7 (18.9)	0.35^b^

*CAPS-CA* Clinician-Administered PTSD Scale for Children and Adolescents, *D-CPT* developmentally adapted cognitive processing therapy, *DSM-IV* Diagnostic and Statistical Manual of Mental Disorders, Fourth Edition, *PTSD* posttraumatic stress disorder

* *p* < 0.05

^a^Overall number of respondents was *n* = 53, but for *n* = 2 respondents no additional study characteristics could be obtained. ^b^Calculated from 2-sided Pearson χ2 test. ^c^Calculated from 2-sided unpaired t test. ^d^Calculated from 2-sided Fisher’s exact test. ^e^Scores range from 0 to 136, with higher scores indicating greater severity of symptoms. ^f^*N* = 65 at 3-month follow up, with *n*_respondents_ = 45 and *n*_non-respondents_ = 20. ^g^Change scores range from 0 to 136, with higher change scores indicating greater reduction of symptoms. ^h^Includes nicotine dependence and borderline personality disorder. ^i^*N* = 66 at 3-month follow up, with *n*_respondents_ = 46 and *n*_non-respondents_ = 20

There were no significant differences between respondents and non-respondents with regard to most sociodemographic characteristics, trauma history or outcome variables concerning PTSD or comorbid disorders at baseline and 3-month follow-up. However, the non-respondents received significantly more support from youth welfare services (prevalence 7 [13.7%] vs. 14 [37.8%]).

### Recruitment source

Most respondents (82.4%) reported multiple recruitment sources with an average of *M* = 3.20, *SD* = 2.0. The recruitment sources reported most frequently were internet search (indicated by 43.1%), study website (reported by 39.2%) and flyers (indicated by 31.4%). Table 2 gives the frequencies of each referral source. The recruitment sources reported least frequently were Facebook, gynecologists, newspaper ads, police departments, posters, school counselors/school psychologists, teachers (indicated by 1 respondent each) and local health offices (not indicated by any respondent). No significant differences between adolescent and young adult respondents were observed, with the exception that adolescents reported significantly more often that they had learned about the trial from their parent/caregiver (prevalence 8 [32.0%] vs. 2 [7.7%]). Overall, most mentions could be categorized as online and media advertising search strategies (74 of 163 mentions [45.4%] in total, including internet search, websites, flyers, posters, mailing lists, newspaper articles and advertisements, and Facebook), followed by practitioner-referred sources (63 of 163 mentions [38.7%] in total, including therapists, physicians, clinics, counselors, social workers, and teachers). A third category corresponded to personal referrals (21 mentions [12.9%], including parents/caregivers, other patients, and close friends).

**Table 2:** Frequencies (%) of reported recruitment sources

Recruitment source^a^	Respondents			*p* value
	All (*N* = 51^b^)	Adolescents aged 14–17 (*n* = 25)	Young adults aged 18–21 (*n* = 26)	
Internet search	22 (43.1)	9 (36.0)	13 (50.0)	0.31^c^
Study website	20 (39.2)	10 (40.0)	10 (38.5)	0.91^c^
Flyer	16 (31.4)	10 (40.0)	6 (23.1)	0.19^c^
Psychiatrist	13 (25.5)	8 (32.0)	5 (19.2)	0.30^c^
Psychotherapist	11 (21.6)	6 (24.0)	5 (19.2)	0.68^c^
Parent/caregiver	10 (19.6)	8 (32.0)	2 (7.7)	0.04^d^*
Other patients	9 (17.6)	5 (20.0)	4 (15.4)	0.73^d^
Social worker	8 (15.7)	5 (20.0)	3 (11.5)	0.47^d^
Website of respective study site	8 (15.7)	3 (12.0)	5 (19.2)	0.70^d^
Counseling center	6 (11.8)	2 (8.0)	4 (15.4)	0.67^d^
Other^e^	4 (7.8)	0 (0)	4 (15.4)	0.11^d^
Outpatient clinic	4 (7.8)	2 (8.0)	2 (7.7)	1.00^d^
Psychiatric clinic	4 (7.8)	3 (12.0)	1 (3.8)	0.35^d^
General hospital	3 (5.9)	2 (8.0)	1 (3.8)	0.61^d^
Pediatrician	3 (5.9)	2 (8.0)	1 (3.8)	0.61^d^
Psychosomatic clinic	3 (5.9)	3 (12.0)	0 (0)	0.11^d^
Students’ mailing list	3 (5.9)	0 (0)	3 (11.5)	0.24^d^
Youth welfare office	3 (5.9)	2 (8.0)	1 (3.8)	0.61^d^
Close friend^e^	2 (3.9)	1 (4.0)	1 (3.8)	1.00^d^
General practitioner	2 (3.9)	1 (4.0)	1 (3.8)	1.00^d^
Newspaper article	2 (3.9)	1 (4.0)	1 (3.8)	1.00^d^
Facebook	1 (2.0)	0 (0)	1 (3.8)	1.00^d^
Gynecologist	1 (2.0)	1 (4.0)	0 (0)	0.49^d^
Newspaper advertisement	1 (2.0)	0 (0)	1 (3.8)	1.00^d^
Police department	1 (2.0)	0 (0)	1 (3.8)	1.00^d^
Poster	1 (2.0)	0 (0)	1 (3.8)	1.00^d^
School counselor/school psychologist	1 (2.0)	1 (4.0)	0 (0)	0.49^d^
Teacher	1 (2.0)	0 (0)	1 (3.8)	1.00^d^
Local health office	0 (0)	0 (0)	0 (0)	Na

*Na* not applicable

* *p* < 0.05

^a^Assessed with the checklist for recruitment sources (see Additional file 1). ^b^Overall number of respondents was *n* = 53, but data from *n* = 2 respondents had to be excluded because of missing information. ^c^Calculated from 2-sided Pearson χ2 test. ^d^Calculated from 2-sided Fisher’s exact test. ^e^Category added post hoc according to free responses

### Barriers to study participation

Forty-three respondents (84.3%) reported some type of barrier to study participation, with an average of *M* = 2.27, *SD* = 1.7, different barriers. The respondents’ most frequently reported barriers were commuting time to the respective study site (indicated by 47.1% of all respondents), volume of questionnaires (35.3%), distress caused by having to talk about painful topics (35.3%) and duration of study appointments (35.4%). Table 3 gives the frequencies of all reported barriers of the respondents. With regard to reported barriers, there were no significant differences between adolescent and young adult respondents. Overall, most respondents reported additional demands of the trial such as transport problems, diagnostic procedures, and video recordings (70 out of 116 mentions [60.3%]).

**Table 3:** Frequencies (%) of reported barriers

Barriers to participation^a^	Respondents			*p* value
	All (*N* = 51^b^)	Adolescents aged 14–17 (*n* = 25)	Young adults aged 18–21 (*n* = 26)	
Commuting time to study site	24 (47.1)	14 (56.0)	10 (38.5)	0.21^c^
Volume of questionnaires	18 (35.3)	10 (40.0)	8 (30.8)	0.49^c^
Distress caused by having to talk about painful topics	18 (35.3)	8 (32.0)	10 (38.5)	0.63^c^
Duration of study appointments	18 (35.3)	11 (44.0)	7 (26.9)	0.20^c^
Alternating interviewers	11 (21.6)	4 (16.0)	7 (26.9)	0.34^c^
Distress caused by fears about the results of diagnostics	9 (17.6)	2 (8.0)	7 (26.9)	0.14^d^
Video recordings	9 (17.6)	4 (16.0)	5 (19.2)	1.00^d^
Difficulties in scheduling appointments^e^	3 (5.9)	1 (4.0)	2 (7.7)	1.00^d^
Other^e^	3 (5.9)	0 (0)	3 (11.5)	0.24^d^
Distress caused by using public transport to the study site^e^	2 (3.9)	1 (4.0)	1 (3.8)	1.00^d^
Reachability of study site	1 (2.0)	0 (0)	1 (3.8)	1.00^d^
Concerns about confidentiality	0 (0)	0 (0)	0 (0)	Na

*Na* not applicable

^a^Assessed with the checklist for barriers to study participation (see Additional file 1). ^b^Overall number of respondents was *n* = 53, but data from *n* = 2 respondents had to be excluded because of missing information. ^c^Calculated from 2-sided Pearson χ2 test. ^d^Calculated from 2-sided Fisher’s exact test. ^e^Category added post hoc according to free responses

### Facilitators for study participation

Fifty (98.0%) respondents reported some type of facilitator for participation in the trial. On average, *M* = 3.27, *SD* = 1.9 facilitators were reported. Flexible time scheduling (reported by 62.7%), financial compensation for attending assessments (52.9%), consistent contact persons (43.1%) and interviewers (39.2%), and social support from friends and relatives (37.3%) were the most frequently indicated facilitators. Again, there were no differences in frequencies of reported facilitators between adolescent and young adult respondents. Altogether, facilitators concerning the organizational setting (i.e., flexible time scheduling, consistent contact persons and interviewers, reminders of appointments, involvement of caregivers; accounting for 92 of 167 mentions [55.1%]) and monetary incentives (including financial compensation for assessments and reimbursement of travel costs; accounting for 37 mentions [22.2%]) were reported most frequently, followed by social support (20 mentions [12.0%]) and non-monetary incentives (i.e., thank-you cards, empathy of study staff, and treatment certificate; 17 mentions [10.2%]). Table 4 gives the frequencies of all indicated facilitating factors.

**Table 4:** Frequencies (%) of reported facilitators

Facilitator for participation^a^	Respondents			*p* value
	All (*N *= 51^b^)	Adolescents aged 14–17 (*n* = 25)	Young adults aged 18–21 (*n* = 26)	
Flexible time scheduling	32 (62.7)	13 (52.0)	19 (73.1)	0.12^c^
Financial compensation for taking part in assessments	27 (52.9)	13 (52.0)	14 (53.8)	0.90^c^
Consistent and reliable contact person at the respective study site	22 (43.1)	9 (36.0)	13 (50.0)	0.31^c^
Same interviewer	20 (39.2)	11 (44.0)	9 (34.6)	0.49^c^
Social support by friends and relatives	19 (37.3)	12 (48.0)	7 (26.9)	0.12^c^
Thank-you cards	12 (23.5)	6 (24.0)	6 (23.1)	0.94^c^
Reimbursement of travel costs	10 (19.6)	4 (16.0)	6 (23.1)	0.73^d^
Involvement of caregiver	9 (17.6)	6 (24.0)	3 (11.5)	0.29^d^
Reminders of appointments	9 (17.6)	2 (8.0)	7 (26.9)	0.14^d^
Empathy of study staff^e^	4 (7.8)	1 (4.0)	3 (11.5)	0.61^d^
Other^e^	2 (3.9)	0 (0)	2 (7.7)	0.49^d^
Certificate at the end of treatment	1 (2.0)	0 (0)	1 (3.8)	1.00^d^

^a^Assessed with the checklist for facilitators for study participation (see Additional file 1). ^b^Overall number of respondents was *n* = 53, but data from *n* = 2 respondents had to be excluded because of missing information. ^c^Calculated from 2-sided Pearson χ2 test. ^d^Calculated from 2-sided Fisher’s exact test. ^e^Category added post hoc according to free responses

## Discussion

### Main findings

This study examined recruitment methods, barriers, and facilitators for study participation reported by adolescents and young adults aged 14 to 21 with PTSD after histories of sexual and/or physical abuse. To this end, the study participants in a RCT evaluating D-CPT were surveyed after having completed the study. We found that most respondents reported multiple recruitment sources, with online and media advertising search strategies indicated most frequently (indicated by 45.4% of all mentions), followed by practitioner-referred recruitment sources (38.7%). A minority of respondents indicated that they had used personal referrals (12.9%). Adolescent respondents (aged 14 to 17) reported significantly more often than young adults (aged 18 to 21) that they had learned about the study from their parent/caregiver. Apart from that, there were no significant differences in reported recruitment sources between age groups. With respect to barriers to participation in this RCT, most respondents reported additional demands of the trial like transport problems, diagnostic procedures, and video recordings (60.3% of all mentions). Furthermore, the distress caused by having to talk about painful topics (15.5% of all mentions) seems to be a specific barrier relevant to trauma history indicated by the respondents. Most of the respondents reported facilitators concerning the organizational setting (e.g., flexible time scheduling, consistent contact persons and interviewers, reminders of appointments, and involvement of caregivers; 55.1% of all mentions) and monetary incentives (22.2%), followed by social support (12.0%) and non-monetary incentives (e.g., thank-you cards; 10.2%).

### Comparison with the literature

Our respondents reported online and media advertising search strategies most frequently. Referrals by practitioners were substantial as well, but in contrast to data from depressive and anorectic adolescents [27, 28] they were not the most frequently reported. This might be attributable to the great variety of online and self-education means applied during the recruitment phase of the trial. Furthermore, some abused adolescents and young adults might prefer to search for help on their own before entrusting themselves personally to a professional. However, given the small number of existing studies analyzing recruitment sources for mental health trials in adolescents [27, 28], it is unclear if our results are specific to the overall age group or to adolescents and young adults suffering from abuse-related PTSD. With regard to Facebook, only one respondent reported Facebook as a recruitment source in the current sample in contrast to prior studies stressing the potential of social media [29]. As most of the existing studies [29] used paid advertising on Facebook and conducted online surveys without the need for in-person responses, comparability with our recruitment methods and study design is not possible. Future work should examine the specific role of paid advertising on social media for on-site RCTs.

The barriers reported by respondents in the current study resembled some of the barriers reported in previous studies with adult participants, such as additional demands on the patient made by the trial [10], fear of stigma (i.e., distress caused by fears about the results of diagnostics, [22]), and trauma-specific barriers (i.e., distress caused by having to talk about painful topics, [22]). Surprisingly, not one respondent indicated concerns about confidentiality as a barrier. But these concerns may have been mitigated by a very detailed and apparently easily understandable oral and written explanation of the study that each study participant received prior to giving assent/consent.

The facilitators reported by respondents in the current study might serve as first recommendations for this clientele as this is the first study on the participation of adolescents and/or young adults with abuse-related PTSD in RCTs. Our results highlight the importance of the special organizational characteristics of the trial for the respondents, especially flexible time scheduling, consistent contact persons and interviewers, and monetary incentives. Social support and non-monetary incentives (i.e., thank-you cards) throughout the trial were facilitative as well.

Adolescents and young adults only differed with regard to the relevance of recruitment via parents/caregivers. This difference was to be expected, as adult participants might decide to seek treatment more autonomously without discussing their problems beforehand with their parents. Surprisingly, there were no further differences between adolescent and young adult respondents with regard to reported recruitment source, barriers, or facilitators. One possible explanation for this might be the German healthcare system. In Germany, youth up to 21 years may be treated by child and adolescent therapists. This may also lead to a comparable relevance of certain recruitment strategies, barriers, and facilitators for adolescents and young adults. A second possible explanation might be that similar developmental tasks have to be mastered equally by adolescents and young adults and this might influence help-seeking behavior as well as perceived barriers and facilitators. Therefore, it might be helpful for recruitment and retention strategies for young adults to be more similar to those for adolescents than to those for older adults. However, in order to clearly confirm these assumptions, further research on effective recruitment and retention strategies across different age groups is urgently needed.

### Strengths and limitations

This study is the first one to generate data on recruitment methods, barriers, and facilitators of specific relevance for young individuals with abuse-related PTSD participating in a RCT. Unlike most studies in the field, we extended the participants’ age range to 21. The inclusion of young adults up to age 21 is in accordance with the German healthcare system, where youth up to 21 years may be treated by child and adolescent therapists. Consequently, extending the age range to 21 strengthens external validity.

Besides these strengths, several limitations need to be acknowledged. First, the generalizability of our study results is limited by the small sample size and the predominantly female (85.2%) sample. Furthermore, we do not know whether the results are also transferable to study participants from other countries due to differences in health care systems. Second, using a methodological approach based on self-report measures might reduce the validity of the results as well as the insight in possible recruitment sources, additional barriers, and facilitators not listed on the checklist. Third, the retrospective design of the study with only study participants being surveyed and without any control condition lessens the strength of the conclusions that can be drawn from the findings. This design did not allow us to compare the effectiveness of individual recruitment strategies or the indirect effects of the applied strategies on respondents’ parents or legal guardians. Moreover, we cannot report on barriers experienced by non-participants or participants who did not respond to our survey. All the same, the response rate of 66.3% was acceptable, although the overall sample size of the trial was rather small.

Both the prospective evaluation of different recruitment strategies and the assessment of distinct barriers experienced by non-participants would, however, help to develop better strategies to facilitate access to both interventions within RCTs and mental health care in general. They should, therefore, be addressed in future studies.

## Conclusions

Our findings are of interest for professionals working with abused young people and permit the formulation of some recommendations when planning trials. The results of our survey indicated that adolescents and young adults suffering from abuse-related PTSD used multiple recruitment sources, with online and media advertising search strategies indicated most frequently. This highlights the importance of relying on a multitude of sources when recruiting this clientele, as even young individuals seem to be interested in obtaining information about the treatment and/or the trial via various means before deciding to contact the study team. Contrary to the procedure in the current trial, it would seem advisable to assess the specific recruitment source of each individual interested in the study, and to make regular interim evaluations of each recruitment source, in order to gain insight into successful and non-successful recruitment strategies at an early stage of the recruitment phase, and to adapt the strategies accordingly, if necessary. Consequently, the specific characteristics of the local study site can be taken into account for further recruitment efforts in due time.

Based on the results on respondents’ reported barriers and facilitators, we believe that some barriers perceived by young individuals might be prevented by planning and organizing the trial accordingly. Consequently, the following seem to be helpful factors for young respondents in order to commit to a trial without terminating: flexibility of appointments, reduced additional demands of the trial, such as the volume of diagnostics, continuity of study personnel in direct contact with the study participants, and financial incentives.

To address trauma-specific barriers, the early dissemination of psychoeducative information about PTSD and avoidance symptoms, as well as the therapeutic rationale, should play an important role when providing information about a trauma-focused trial, for example in the study clarification process. To reduce concerns about the study, detailed and age-adapted explanations of the study might help to overcome potential barriers.

With some limitations, our results also may allow to be extrapolated to retention strategies to clinical treatment with this population in general. Therefore, we would like to give some clinical recommendations for therapeutic work addressing the barriers identified by respondents in this study, which can be obtained in Table 5. Given the small evidence base for facilitators regarding therapeutical work in adolescents and young adults suffering from PTSD, these considerations should be understood as recommendations stemming from clinical practice. However, based on our clinical experience, consideration of these aspects is crucial when working with adolescents and young adults suffering from PTSD.

**Table 5:** Clinical recommendations for addressing respondents’ reported barriers

Barrier reported by respondents	Recommended facilitator
Commuting time to therapy center; distress caused by using public transport to therapy center; reachability of study site	Fostering and providing internet-based or mobile-based interventions
Volume of questionnaires, duration of diagnostic appointments	Providing detailed information on aims and purposes of each assessment measure; providing decision-making options with regard to splitting parts of diagnostics on several appointments; providing detailed feedback on the results
Distress caused by having to talk about painful topics	Providing detailed psychoeducation on common patients‘ reactions to and risks and benefits of diagnostics; providing psychoeducation on PTSD symptoms and especially avoidance symptoms
Alternating interviewers	Ensuring continuity of interviewers
Distress caused by fears about the results of diagnostics	Elaborating possible risks and benefits of getting a mental health diagnosis
Video recordings	Providing detailed information on aims and purposes of video recordings; providing decision-making options
Difficulties in scheduling appointments	Offering flexible time scheduling
Concerns about confidentiality	Providing detailed information on legal and ethical requirements and possibilities

In conclusion, this is the first study to analyze recruitment sources, barriers, and facilitators for adolescent and young adult study participants with abuse-related PTSD. To confirm the validity of our results, future research should focus on the efficacy of certain recruitment sources of specific relevance for this clientele and on how specific barriers can be overcome effectively. This might also be helpful in identifying strategies to overcome general barriers to help-seeking behavior in young individuals suffering from PTSD and to facilitate therapeutic care.

## Supplementary information

**Additional file 1:** Overview on recruitment and retention strategies applied during the D-CPT trial.

**Additional file 2:** Survey on experiences in the D-CPT trial. Checklist to assess participants’ individual recruitment sources, barriers and facilitators for study participation, English translation.

## Data Availability

The datasets analyzed during the current study are available from the corresponding author upon reasonable request.

## Abbreviations

CAPS-CA: Clinician-Administered PTSD Scale for Children and Adolescents
D-CPT: Developmentally adapted Cognitive Processing Therapy
DSM-IV: Diagnostic and Statistical Manual of Mental Disorders, Fourth Edition
IRB: Institutional Review Board
PTSD: Posttraumatic stress disorder
RCT: Randomized controlled trial
SCID-I: Structured Clinical Interview for DSM-IV Axis I
WL/TA: Wait-list condition with treatment advice
