# Supplementary material for: Challenges in recruiting and retaining adolescents with abuse-related posttraumatic stress disorder: lessons learned from a randomized controlled trial
Source: Child Adolesc Psychiatry Ment Health. 2020 Apr 16;14:14. doi: 10.1186/s13034-020-00320-y (PMC7164245; doi:10.1186/s13034-020-00320-y)
Supplement: Supplementary file 1 — Additional file 1: Overview on recruitment and retention strategies applied during the D-CPT trial. [file 13034_2020_320_MOESM1_ESM.pdf]

**Overview on recruitment and retention strategies applied during the D-CPT trial**

## Recruitment Strategies

**Electronic, radio, and print media advertising.** During the recruitment phase (09/2012 –05/2015) seven press releases of the involved university outpatient clinics were distributed by the study sites. As a result, several local newspapers contacted the study coordinators or the principal investigator for interview requests on the topic and the study. Furthermore, the coordinators were instructed to proactively contact local newspapers and magazines and provide them with topic information and interviews. Over the recruitment phase, 17 interviews and articles on the trial were published in local print newspapers and online magazines. One interview was broadcast by a local radio station. Advertisements in local print newspapers and online platforms were posted in compliance with the recruitment and advertising guidelines of the respective university. Newspaper advertisements were placed in the Classified sections of popular local newspapers. Online advertisements were posted on a study website advertised by Google AdWords, on the respective websites of the three study sites, on a social media platform (Facebook), on advertisement websites such as eBay Classifieds, and on internet advertising portals for psychotherapists.

**Community outreach and institution referral.** Regular appearances at conferences and network meetings of the local health care and social/youth welfare community were used to inform the local community about our trial. Other outreach activities included talks on our project given by the principal investigator or the study coordinators to various community groups (e.g., schools, clinics, police departments). Nine articles on the study project were published in journals and magazines of German psychotherapeutic, medical, and school professional associations during the recruitment phase. To further increase community outreach efforts and establish referral networks, we sent information letters and emails with

attached flyers to institutions involved in local (mental) health: psychiatric, psychosomatic and outpatient psychiatric and psychotherapeutic clinics, general hospitals, counseling centers, psychiatrists, psychotherapists, pediatricians, gynecologist, general practitioners in own practice, local health offices, local branches of psychotherapeutic and medical professional associations, and health insurance companies.

With respect to the youth and social welfare community, we contacted local youth and social welfare offices, women's refuges, facilities run by the German child and adolescent welfare agency, and youth centers.

We also sent information letters and flyers to all secondary schools in the vicinity of the respective study sites, to school psychologists, and school counselors. To inform the local university community, we contacted student counseling offices, and distributed information on the project via students' mailing lists and posters in university buildings.

In addition, we informed the local communities by posters on our project distributed in busses and shops near schools.

**Word-of-mouth.** Word-of-mouth recruitment occurred through individuals who knew about our research (e.g., through media coverage, flyers) or former participants. We did not directly invite participants to suggest the study to friends who might also be in need of trauma-focused therapy.

**Retention Strategies**

To enhance retention in the trial, we used identical strategies at each study site. Monetary incentives included financial compensation for completing assessments (i.e., €20 for midtreatment and posttreatment assessments, €30 for 3-month follow-up assessments) and reimbursement of travel costs for assessments and therapy sessions, if the participant and, in the case of minors, the caregivers could not afford to pay for bus/train tickets themselves. Non-monetary incentives included thank-you cards, which were used in letters to remind

participants of their next assessment appointments. Furthermore, participants received a certificate at the end of treatment confirming their successful completion of the therapy program.

Study staff were instructed to show understanding and patience in their encounters with all study participants and to offer meeting times that were as flexible as possible. Study sites were also instructed to ensure continuity by defining consistent contact persons and having the same interviewer conduct the repeated assessments, as far as this was compatible with rater blinding.
