# Supplementary material for: Challenges in recruiting and retaining adolescents with abuse-related posttraumatic stress disorder: lessons learned from a randomized controlled trial
Source: Child Adolesc Psychiatry Ment Health. 2020 Apr 16;14:14. doi: 10.1186/s13034-020-00320-y (PMC7164245; doi:10.1186/s13034-020-00320-y)
Supplement: Supplementary file 2 — Additional file 2: Survey on experiences in the D-CPT trial. Checklist to assess participants’ individual recruitment sources, barriers and facilitators for study participation, English translation. [file 13034_2020_320_MOESM2_ESM.pdf]

Traumatherapie für Jugendliche · E-KVT

Survey on experiences in the D-CPT trial

1. **How did you become aware of the D-CPT trial? Please tick all applicable answers and add your answer below, if you can't find it in the list. Several answers are possible.**

O Recommendation by another participant

O Recommendation by a psychiatrist

O Recommendation by a general practitioner

O Recommendation by a pediatrician

O Recommendation by a gynecologist

O Recommendation by a school psychologist or school counselor

O Recommendation by a teacher

O Recommendation by a social worker

O Recommendation by the youth welfare office

O Recommendation by the local health office

O Recommendation by a police department

O Internet search (e.g., via Google)

O Website of the study

O Website of the study centre

O Facebook

O Other(s), namely:

O Flyer

O Advertisement in a newspaper

O Article in a newspaper

O Poster

O Recommendation by psychotherapists

O Recommendation by staff of an outpatient clinic

O Recommendation by staff of a psychiatric clinic

O Recommendation by staff of a psychosomatic clinic

O Recommendation by staff of a general hospital

O Recommendation by a counseling center

O Receiving an e-mail via a students' mailing list

O Recommendation by my parents/ guardians

____

____

____

Additional file 2. Vogel, A., Comtesse, H., Rosner, R. Challenges in recruiting and retaining adolescents with abuse-related posttraumatic stress disorder: lessons learned from a randomized controlled trial.

2. **What were the problems for you to participate in the D-CPT trial? Please tick all applicable answers and add your answer below, if you can't find it in the list. Several answers are possible.**

O Duration of assessment appointments
O Distress caused by fearing the results of the diagnostics
O Commuting time to the study site
O Difficult reachability of study site
O Amount of questionnaires to be completed
O Other(s), namely:
O Distress caused by having to talk about painful topics
O Alternating interviewers
O Video recordings
O Concerns about confidentiality

____

____

____

3. **What was helpful to participate in the D-CPT trial?**

O Financial compensation for taking part in assessments
O Reimbursement of travel costs
O Thank you cards
O Certificate after successful completion of the therapy
O Flexible time scheduling
O Other(s), namely:
O Involvement of parents/caregivers
O Consistent interviewer at most appointments
O Consistent contact person at the study site
O Reminders of appointments
O Support by friends and/or relatives

____

____

____

Thank you very much for answering these questions, you've helped us a lot!
